# Hemangiomas and Vascular Malformations: Current Theory and Management

**DOI:** 10.1155/2012/645678

**Published:** 2012-05-07

**Authors:** Gresham T. Richter, Adva B. Friedman

**Affiliations:** Division of Pediatric Otolaryngology, Department of Otolaryngology-Head and Neck Surgery, University of Arkansas for Medical Sciences, Arkansas Children's Hospital, 1 Children's Way, Little Rock, AR 72202, USA

## Abstract

Vascular anomalies are a heterogeneous group of congenital blood vessel disorders more typically referred to as birthmarks. Subcategorized into vascular tumors and malformations, each anomaly is characterized by specific morphology, pathophysiology, clinical behavior, and management approach. Hemangiomas are the most common vascular tumor. Lymphatic, capillary, venous, and arteriovenous malformations make up the majority of vascular malformations. This paper reviews current theory and practice in the etiology, diagnosis, and treatment of these more common vascular anomalies.

## 1. Introduction

Vascular anomalies are congenital lesions of abnormal vascular development. Previously referred to as vascular birthmarks, vascular anomalies are now classified based on a system developed in 1982 by Mulliken and Glowacki that considers histology, biological behavior, and clinical presentation of these entities [1]. A primary distinction is made between a vascular tumor, which grows by cellular hyperplasia, and a vascular malformation, which represents a localized defect in vascular morphogenesis. Due to the differences in biologic and radiographic behavior, malformations are further divided into slow-flow and fast-flow lesions (Table 1).

Both vascular tumors and malformations may occur anywhere on the body. In brief, hemangiomas are vascular tumors that are rarely apparent at birth, grow rapidly during the first 6 months of life, involute with time and do not necessarily infiltrate but can sometimes be destructive. Vascular malformations are irregular vascular networks defined by their particular blood vessel type. In contrast to hemangiomas, they are present at birth, slow growing, infiltrative, and destructive. Almost all vascular malformations and nearly 40% of hemangiomas eventually require intervention. Thus, this paper offers pediatricians an update on recent developments in the diagnosis, management, and pathogenesis of vascular anomalies. Due to their complexity, a multidisciplinary approach is frequently necessary in managing these lesions and includes a team of specialists in pediatric otolaryngology, dermatology, hematology, interventional radiology, surgery, orthopedics, and sometimes psychology.

## 2. Hemangiomas

Infantile hemangiomas are the most common tumor in infancy and occur in approximately 10% of the population. Identifiable risk factors include female sex, prematurity, low birth weight, and fair skin [2]. They consist of rapidly dividing endothelial cells. Because their growth is attributed to hyperplasia of endothelial cells, they are classified as, and are the most common, vascular tumors.

Hemangiomas are further categorized into two types: “infantile” or “congenital.” The rare “congenital” hemangioma is less understood and present at birth. Congenital hemangiomas either rapidly involute (rapidly involuting congenital hemangioma (RICH)) over a very brief period in infancy or never involute (noninvoluting congenital hemangioma; (NICH)). The remaining sections will focus on the more common “infantile” hemangiomas.

The pathogenesis of infantile hemangiomas remains unclear, although two theories dominate current thought. The first theory suggests that hemangioma endothelial cells arise from disrupted placental tissue imbedded in fetal soft tissues during gestation or birth. Markers of hemangiomas have been shown to coincide with those found in placental tissue [3]. This is further supported by the fact that they are found more commonly in infants following chorionic villus sampling, placenta previa, and preeclampsia [2]. A second theory arose from the discovery of endothelial progenitor and stem cells in the circulation of patients with hemangiomas [4]. The development of hemangiomas in animals from stem cells isolated from human specimens supports this theory [5]. However, infantile hemangiomas most likely arise from hematopoietic progenitor cells (from placenta or stem cell) in the appropriate milieu of genetic alterations and cytokines. Abnormal levels of matrix metalloproteinases (MMP-9) and proangiogenic factors (VEGF, b-FGF, and TGF-beta 1) play a role in hemangioma pathogenesis [6]. Genetic errors in growth factor receptors have also been shown to affect development of hemangiomas [7].

### 2.1. Diagnosis

**Figure 1:**
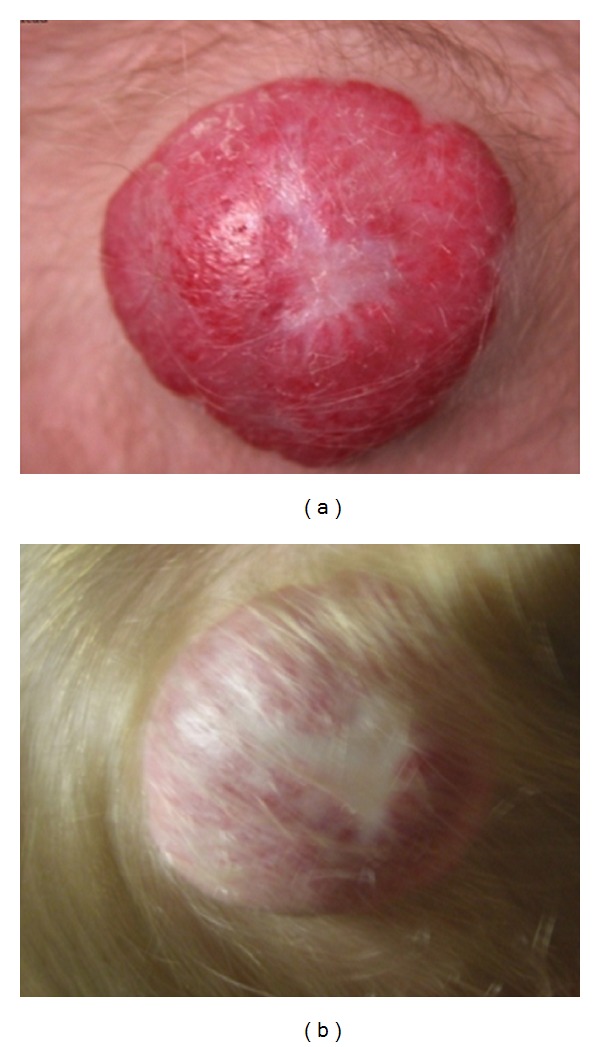
(a) Proliferating hemangioma at 3 months of age. (b) Same hemangioma at involution at 4 years of age.

Infantile hemangiomas present shortly after birth most often as well-demarcated, flat, and erythematous red patches. At this stage, hemangiomas may be confused with other red lesions of birth, but rapid proliferation and vertical growth will trigger the diagnosis (Figure 1(a)). Generally speaking, hemangiomas do not spread outside their original anatomical boundaries. Hemangiomas follow a predictable course with three distinct developmental phases: proliferation, quiescence, and involution. In most hemangiomas, eighty percent of proliferation occurs by three months of life but may last longer [8]. During proliferation, rapid growth can lead to exhaustion of blood supply with resulting ischemia, necrosis, ulceration, and bleeding.

Hemangiomas can be superficial, deep, or compound. The superficial hemangioma is red and nodular with no subcutaneous component. A deep hemangioma presents as a protrusion with an overlying bluish tint or telangectasia. Compound hemangiomas have both deep and superficial components (Figure 1(b)). This new nomenclature helps eliminate confusing older terms (Table 2). 

Following proliferation, hemangiomas enter a slower or no growth phase, known as quiescence. This phase typically lasts from 9 to 12 months of age. The final and unique phase of the hemangioma lifecycle is involution. This phase is marked by graying of the overlying skin and shrinking of the deeper components (Figure 1(b)). Historical reports suggest that involution of 50%, 70%, and 90% of the hemangioma occurs by 5, 7, and 9 years of age with some variability [9]. At the final stages of involution, a fibrofatty protuberance may remain (Figure 1(b)).

**Figure 2:**
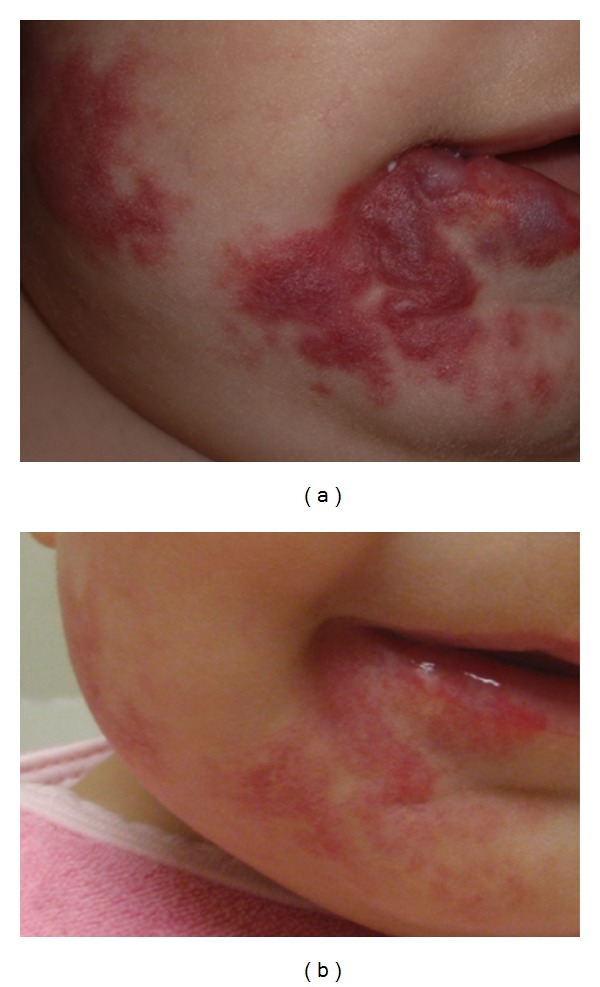
(a) Segmental hemangioma in trigeminal (V3) distribution. (b) Same hemangioma after 2 months of therapy with propranolol (2 mg/kg divided tid).

Another subclassification for hemangiomas is focal versus segmental disease. Focal hemangiomas are localized, unilocular lesions which adhere to the phases of growth and involution. Multifocal hemangiomatosis also exists, and infants with greater than 5 lesions should undergo workup to rule out visceral involvement. Segmental hemangiomas are more diffuse plaquelike and can lead to untoward functional and aesthetic outcomes. The limb and face are common locations for disease (Figure 2). Head and neck lesions frequently coincide with the distribution of the trigeminal nerve. A beard-like distribution is associated with a subglottic hemangioma 60% of the time [10]. Regardless, a stridulous child with either a focal or segmental hemangioma should be presumed to have subglottic disease until proven otherwise.

Patients with segmental hemangiomas should also undergo investigation to rule out PHACES syndrome (posterior fossa brain malformations, hemangiomas of the face, arterial cerebrovascular anomalies, cardiovascular anomalies, eye anomalies, and sternal defects or supraumbilical raphe) [11]. 

The diagnosis of a hemangioma is best made by clinical history and physical exam. In cases of unclear diagnosis, the best radiographic modalities to use are either a Doppler ultrasound or MRI.

### 2.2. Management

Historically, hemangiomas have been managed with close observation over their lifecycle [9]. However, research suggests that nearly 40% of children require further intervention because of bleeding, ulceration, visual axis obstruction, airway obstruction, high-output cardiac failure, or risk for permanent disfigurement [12]. With novel therapeutic options as well as a better understanding of disease, observation is declining as the sole means of treating hemangiomas. Nonetheless, inconspicuous lesions are still best treated with observation alone.

Medical and surgical options are available for the treatment of “problematic” hemangiomas. Medical management includes one or more systemic therapies. Corticosteroids, interferon, and vincristine have been successful for massive and life-threatening disease [13–15]. These agents have also been used for multifocal disease, visceral involvement, segmental distribution, airway obstruction, and periorbital lesions. However, significant side effects accompany systemic therapy and have even led to the rejection of some agents as a treatment option.

Surgical management involves excision, laser treatment or both. Intralesional steroid treatment is also an option for focal hemangiomas of the parotid, nasal tip, subglottis, and eyelid. Repeat therapy is often required, but systemic side effects are limited [16]. 

Excision is the appropriate for localized lesions the fibrofatty remnants (residuum) of involuted hemangiomas. Elective subtotal excision of massive protuberant proliferating hemangiomas can be employed in order to maintain aesthetic facial boundaries. Small remnants of disease are then left for involution. Residual erythema and telangiectasias frequently remain in involuted hemangiomas and are best treated by selective photothermolysis using the flash pulse dye laser (FPDL). Similarly, ulcerative lesions during proliferation can be treated with FPDL to induce healing and new epidermal growth.

### 2.3. Propranolol

A paradigm shift has occurred regarding the treatment of hemangiomas over the past few years. In 2008, propranolol, a nonselective *β*-adrenergic antagonist, was serendipitously discovered to cause regression of proliferating hemangiomas in newborns receiving treatment for cardiovascular disease [17]. Numerous studies demonstrating the success of propranolol for shrinking hemangiomas have followed suit [17–19]. In fact, over ninety percent of patients have dramatic reduction in the size of their hemangiomas as early as 1-2 weeks following the first dose of propranolol (Figure 2(b)). Dosing for propranolol in treating hemangiomas is recommended to be 2-3 mg/kg separated into two or three-times-a-day regimens [20]. These doses are dramatically below the concentration employed for cardiovascular conditions in children. Thus, reported side effects of propranolol for hemangiomas have been minimal. Nonetheless, serious concerns for hypoglycemia and lethargy that can occur with this medicine should not be brushed aside [21, 22]. To address these concerns, parents are instructed to give propranolol with meals, report any unusual sleepiness, and not administer it during infections. Early and frequent visits to assess vital signs are recommended in young infants while on therapy. Exacerbation of gastroesophageal reflux may result due to beta-receptor blockade at the lower esophageal sphincter [18]. 

Monitoring the administration of propranolol varies among institutions and practitioners. A unified approach has not yet been determined. However, elective admission with cardiovascular monitoring may be necessary. Outpatient administration with close monitoring has also been successfully performed [23]. Nonetheless, an electrocardiogram must be reviewed by a pediatric cardiologist prior to administration. Cardiopulmonary conditions at risk for propranolol therapy such as heart block or reactive airway disease should draw careful consideration before administering. Consensus on patient monitoring and best dose regimens remains to be determined, but prospective research is underway.

Propranolol is currently employed for “problematic” hemangiomas, those that would have received either surgical or some other systemic therapy to prevent untoward side effects. Subglottic, periorbital, and massive hemangiomas seem to respond well [24]. Despite the success of propranolol in reducing hemangioma size, adjuvant therapy may be necessary in up to 50% of patients [17]. Propranolol's mechanism on treating hemangiomas remains unclear but may involve the regulation of vascular growth factors and hemodynamic cytokines.

## 3. Vascular Malformations Overview

Vascular malformations are rare vascular anomalies composed of inappropriately connected vasculature. Any blood vessel type, or a combination thereof, can be affected in a vascular malformation. These lesions infiltrate normal tissue which makes them very difficult to manage. The most common vascular malformations include lymphatic malformations (LMs), capillary-venular malformations (CM), venous malformations (VMs) and arteriovenous malformations (AVMs) which have been selected to be covered in this paper (Table 1). While different in their biologic and clinical profile, as a whole, vascular malformations do not regress and continue to expand with time. Periods of rapid growth, infiltration, and soft tissue destruction will spur therapeutic approaches that depend upon the malformation involved.

## 4. Lymphatic Malformations

Lymphatic malformations (LMs) are composed of dilated lymphatic vessels with inappropriate communication, lined by endothelial cells and filled with lymphatic fluid. Their incidence is approximated to be 1 in 2000 to 4000 live births [25]. Lesions are classified as macrocystic (single or multiple cysts >2 cm^3^), microcystic (<2 cm^3^), or mixed [1]. Previous terminology, that is no longer used, has included “cystic hygroma” and “lymphangioma” to describe these entities.

The etiology of LM is unclear. Although most are congenital, there have been reports of LM occurring after trauma or infection. Receptors involved in the formation of lymphatic vascular channels, such as VEGFR3 and Prox-1, may play a role in the development of this disease [26].

### 4.1. Diagnosis

Lymphatic malformations may be macrocystic, microcystic, or mixed. Gradual growth and expansion is typical. Approximately half of the lesions are present at birth and 80–90% by 2 years of age. Local infections approximating the course of lymphatic drainage will cause LM to swell, protrude, and sometimes become painful. This is a hallmark of a LM versus other vascular anomalies that do not present in this fashion.

**Figure 3:**
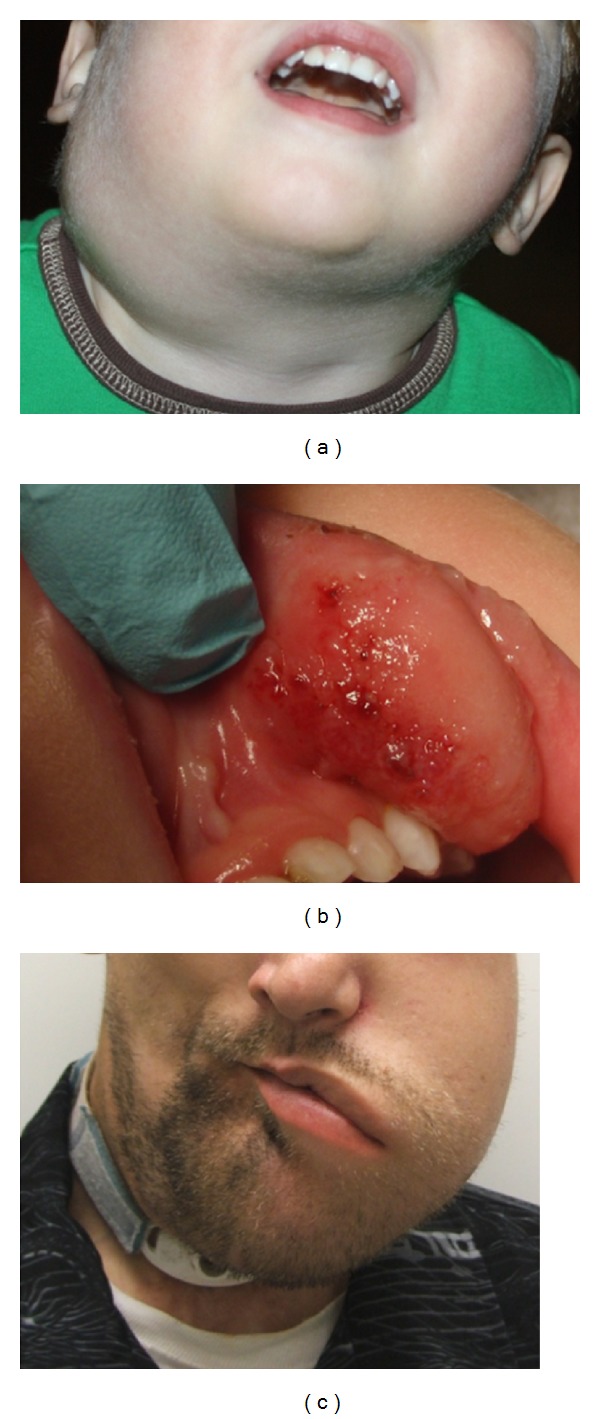
(a) Macrocystic lymphatic malformation (LM) of right neck in toddler. (b) Microcystic lip LM displaying mucosal vesicles. (c) Microcystic LM in older patient with bone involvement and mandibular hypertrophy.

Clinically, the appearance of macrocystic disease differs from that of microcystic. Macrocystic LMs present as a soft, fluid-filled swelling beneath normal or slightly discolored skin (Figure 3(a)). Intracystic bleeding or a mixed lymphatic venous malformation may result in blue discoloration of the overlying skin. Microcystic LMs are soft and noncompressible masses with an overlying area of small vesicles involving the skin or mucosa. These vesicles can weep and at times cause pain or minor bleeding (Figure 3(b)). 

LM can occur anywhere on the body, and symptoms are determined by the extent of disease. Most LMs are found in the cervicofacial region and extend to involve the oral cavity or airway, especially when mixed or microcystic [27]. Symptoms secondary to bulky disease often include pain, dysphagia, odynophagia, impaired speech, or in severe cases, airway obstruction. When involving the skeletal framework in this area, LMs often cause osseous hypertrophy leading to dental or extremity abnormalities (Figure 3(c)).

Although these malformations can usually be diagnosed by physical examination, MRI is used to confirm diagnosis, identify cystic architecture, and determine extent of disease.

### 4.2. Management

An ideal option for treatment of LM does not exist. Several interventions may be required. There have been rare cases of sporadic resolution of a lesion although the majority of these malformations continue to enlarge with age [27]. Macrocystic lesions are more amenable to treatment and have a better prognosis. Swelling from acute infection is best controlled with a short course of systemic steroids and antibiotics. Definitive treatment is delayed until resolution.

LM may be detected on prenatal ultrasound and may require special interventions during delivery. The EXIT (ex utero intrapartum treatment) procedure provides good airway control of the infant if compromise is suspected to occur at birth.

Sclerotherapy is frequently employed for lymphatic malformations, especially if deep seated and difficult to access surgically. It involves injection of a sclerosing agent directly into the lesion leading to fibrosis and ultimately regression of the cysts. Several treatments are usually required, and swelling is expected following therapy. Macrocystic lesions are more easily treated in this fashion, but there have been reports of success in microcystic lesions [28]. Several agents have been utilized for lymphatic malformations including ethanol, bleomycin, OK-432, and doxycycline [29, 30]. Complications include skin breakdown, pain, and swelling. Severe swelling can at times occur and may lead to airway obstruction requiring intensive care [31]. Risks to local nerves are also real but usually result in only transient loss of function.

Carbon dioxide laser therapy may also be employed in limited disease of the airway and oral mucosa [32]. Macrocystic disease is often cured with surgical extirpation. Surgical excision is also frequently employed for microcystic disease although it is more aggressive, invasive, and difficult to control [33, 34]. Infiltration of normal soft tissue and bone by extensive microcystic LM requires massive resections and local or free-flap reconstruction. Failure to completely excise microcystic LM often leads to recurrence. Surgery is also employed in the correction of secondary deformities caused by LM such as bony overgrowth of the facial skeleton [34]. 

Overall, treatment for LM should be aimed at complete elimination of disease. When this is not feasible, multiple treatment modalities are combined to control disease and provide satisfactory functional outcomes.

## 5. Capillary Malformations

**Figure 4:**
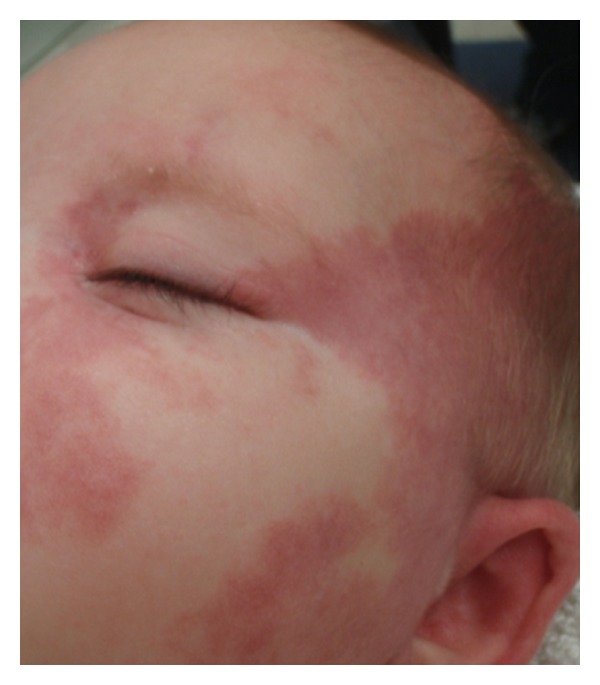
Capillary malformation (port wine stain) of the left face in infant.

Capillary malformations (CMs) are sporadic lesions consisting of dilated capillary-like channels. They occur in approximately 0.3% of children. CMs can present on any part of the body, but are mostly found in the cervicofacial region. They are categorized as medial or lateral lesions depending on their locations. Medial CM gradually lighten with time and eventually disappear. Colloquially they are referred to as stork bites on the nape of the neck and angel kisses on the forehead. Lateral lesions, commonly referred to as port-wine stains, have a more protracted course (Figure 4).

Pathogenesis of isolated capillary malformations is unknown. A genomewide linkage analysis has identified a locus on chromosome 5q associated with familial disease [35]. A rare autosomal dominant inherited disease consisting of a combination of CM and arteriovenous malformations (AVM) is associated with a loss-of-function mutation in RASA1 gene [36]. This has spurred further research into the cause of the more common sporadic form of CM.

### 5.1. Diagnosis

CMs present at birth as flat, red or purple, cutaneous patches with irregular borders. They are painless and do not spontaneously bleed. Lateral CMs, or port-wine stains, usually involve the face and present along the distribution of the trigeminal nerve. CMs tend to progress with time as the vessel ectasia extends to involve deeper vessels to the level of the subcutaneous tissues. This causes the lesion to become darker in color, as well as more raised and nodular [37].

Although they are mostly solitary lesions, CM may exist as a part of a syndrome. The most common of these is the Sturge-Weber syndrome (SWS) and is characterized by a CM in the region of the ophthalmic branch of the trigeminal nerve, leptomeningeal angiomatosis, and choroid angioma. Symptoms of SWS are variable among cases and include intractable seizures, mental retardation, and glaucoma. CM may also be present in Klippel-Trenaunay Syndrome (KTS). This syndrome consists of a combination of multiple lymphatic, venous, and capillary abnormalities.

Diagnosis is usually made by physical examination alone. If here are findings inconsistent with CM exist, for example, pain or spontaneous bleeding, an MRI may be performed. An MRI of the brain as well as an annual ophthalmological exam is warranted when suspicion for SWS is present.

### 5.2. Treatment

The mainstay of treatment for CM is laser therapy. The FPDL is efficacious in treating these lesions. The laser slowly causes the redness of the lesion to fade; therefore, many treatments are often necessary [38]. Early treatment of these lesions appears to slow the progression of the disease. The argon, potassium-titanyl-phosphate (KTP) lasers, and 755 nm laser have also been utilized in more advanced lesions with good outcomes [39]. Surgical excision is also an option in lesions not amenable to laser therapy. This is especially true in advanced lesions which have become nodular [37]. 

## 6. Venous Malformations

Venous malformations (VMs) are slow-flow vascular anomalies composed of ectatic venous channels. These aberrant venous connections lead to venous congestion, thrombosis, and gradual expansion of these lesions. As a result, VMs persist and progress until therapeutic intervention. The incidence of VMs is approximately 1 in 10,000 [40]. VMs more commonly occur sporadically, but research into multifocal disease and familial patterns has helped discover suspected genetic loci involved in their development. There are inherited forms of VMs, the cause of which has been localized to chromosome 9p [41]. Recently a loss-of-function mutation was discovered on the angiopoetin receptor gene TIE2/TEK in many solitary and multiple sporadic venous malformations [42]. In addition, upregulation of several factors including tissue growth factor beta (TGF-beta) and basic fibroblast growth factor (beta-FGF) has been discovered in patients with venous malformations [26]. Progesterone receptors have been discovered in venous malformations. This likely explains their tendency to grow rapidly during hormonal changes [43]. 

### 6.1. Diagnosis

**Figure 5:**
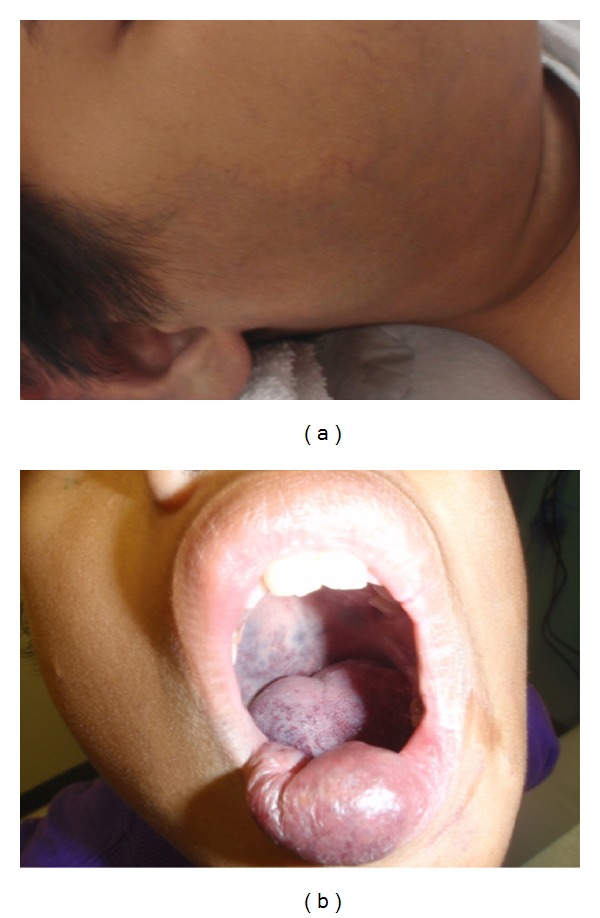
Cervicofacial venous malformation involving the right neck (a) and oropharyngeal mucosa (b).

Venous malformations are often visible at birth but may present as a deep mass. Protrusion may be the only presenting symptom. They are known to grow proportionately with the child with sudden expansion in adulthood. Rapid growth may occur during puberty, pregnancy, or traumatic injury. VM can be either well localized or extensive. The overlying skin may appear normal or possess a bluish discoloration. With more cutaneous involvement, the lesions appear darker blue or purple (Figure 5(a)). Upper aerodigestive involvement is common, and VM are particularly evident when mucosa is affected (Figure 5(b)).

VMs are compressible and swell when the region is dependent or there is an increase in hydrostatic pressure such as during a valsalva maneuver. With time, pain and swelling will occur with the formation of phleboliths (calcified thrombi), or small clots, secondary to trauma or venous stasis. For very large lesions with significant thrombosis the risk of distal emboli remains low but real. D-dimers may be elevated and a marker of disease [44]. When isolated, VM are generally benign with slow growth. They expand secondary to venous stasis and elastic vascular expansion. Airway obstruction, snoring, and sleep apnea may also be present with recumbence [45]. VM can occur anywhere in the body but often are found in the head and neck where they involve the oral cavity, airway, or cervical musculature. MRI is the imaging modality of choice when diagnosing VM and offers superior delineation of disease for treatment planning [46].

### 6.2. Treatment

No single treatment modality is favored in the treatment of VMs and often more than one modality is utilized [47]. Surgery, Nd : YAG laser therapy, and sclerotherapy (directed vascular injury) are all options for treating VM.

Conservative observation of small VM in children may be an option with the knowledge that growth is imminent. Elevating the involved area can decrease hydrostatic pressure and vascular expansion and may impede growth. In large lesions, elevation also decreases swelling and improves pain and airway obstruction. Similarly, compression garments are the initial treatment of choice for advanced limb lesions allowing risks from other treatment options to be avoided. Low-molecular-weight heparin can improve pain from thrombosis [44].

Treatment of larger airway and multifocal disease is often warranted. Symptom-directed therapy is the goal for these lesions. Management techniques typically aim to relieve airway symptoms, pain, and/or disfigurement. Surgical resection and sclerotherapy alone can, at times, be curative for smaller lesions. Local recurrence may occur years after treatment.

Laser therapy provides good control of VM [48]. Use of the Nd : Yag and KTP lasers has been described [47, 49]. The Nd : Yag laser can be used via a fiber attached to an endoscope to treat intraoral and airway venous malformations. Direct injury to deep venous malformations may also be performed by passing the laser directly into the lesion (interstitial therapy). The laser causes shrinking of the lesion along with thrombosis. Serial treatment with these lasers offers reduction and control of disease [48]. Nerve injury may occur with interstitial laser.

Sclerotherapy, as described above, has been used extensively for treatment of VM [50]. The sclerosants most commonly used include ethanol and sotradecol [51]. Complications of sclerotherapy include skin and mucosal injury, swelling leading to airway compromise, infection, and nerve injury. In addition, each sclerosant has its own risk profile. Cardiovascular shock can occur with ethanol, shock-like symptoms with OK-432, interstitial pneumonia or pulmonary fibrosis with bleomycin, and tooth discoloration or electrolyte abnormalities with doxycycline [33]. 

Surgery remains one of the most superior treatment options and may offer a cure for localized VM. Excision of complex lesions remains difficult secondary to intraoperative bleeding. Preoperative sclerosant can be used prior to excision (24–48 hours) to decrease surgical risk. Patients with extensive disease will often require combined modality therapy. Cure is not common, but disease control for many years is often achieved.

## 7. Arteriovenous Malformations

Arteriovenous malformations (AVMs) are congenital high-flow vascular malformations composed of anomalous capillary beds shunting blood from the arterial system to the venous system. They are often misdiagnosed at birth as other vascular lesions because of the delay in presentation of characteristic signs of the malformation. Puberty and trauma trigger the growth of the lesion and manifestation of its troublesome symptoms [52]. They are infiltrative causing destruction of local tissue and often life-threatening secondary to massive bleeding. Extracranial AVMs are different from their intracranial counterpart and are found in several areas in the cervicofacial region.

Little is known about the origin and pathogenesis of AVM. A defect in vascular stabilization is thought to cause AVM, but it remains unclear whether these lesions are primarily congenital in origin. Most AVM, are present at birth, but there are several case reports of these lesions presenting after trauma in adults. Defects in TGF-beta signaling and a genetic two-hit hypothesis are the prevailing theories to the pathogenesis [53, 54]. Progesterone receptors have been isolated in AVMs explaining their expansion during puberty [43].

### 7.1. Diagnosis

**Figure 6:**
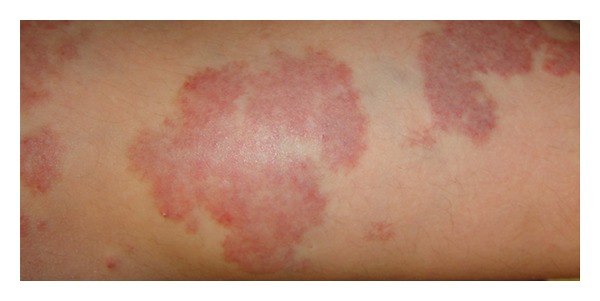
Evidence of skin involvement in limb AVM. Patchy erythematous areas are palpably warmer and pulsatile relative to adjacent skin.

Diagnosis of AVM is based upon clinical examination and imaging. A growing hypervascular lesion may have been present as a slight blush at birth. AVMs are often quiescent for many years and grow commensurate with the child. Intermittent expansion will suggest the diagnosis [52]. Hormonal changes are thought to influence growth [43]. The distinguishing characteristics of an AVM will be palpable warmth, pulse, or thrill due to its high vascular flow [55]. The overlying skin may have a well-demarcated blush with elevated temperature relative to adjacent skin (Figure 6).

The natural course of AVM is early quiescence, late expansion, and ultimately infiltration and destruction of local soft tissue and bone. Common sites for occurence are the midface, oral cavity, and limbs [52]. Oral lesions can present early due to gingival involvement, disruption of deciduous teeth, and profuse periodontal bleeding. Although both focal (small vessel) and diffuse lesions exist, AVMs are by far the most difficult vascular anomaly to manage due to the replacement of normal tissue by disease vessels and very high recurrence rates [55, 56]. 

Imaging is essential in identifying the extent of AVM. MRI may be useful, but MRA and CTA can give a superior outline of these lesions [57]. Numerous hypolucent arterial flow voids are the hallmark of AVM by MRI. CTA allows evaluation of surrounding tissues and bones. Individual arterial feeders can be visualized with this imaging as well [58]. An arteriogram, the time-tested approach to diagnosing AVM, will provide good definition of central “nidus” of affected vessels and provide access for intravascular treatment when necessary [59]. 

### 7.2. Treatment

Treatment of AVM consists of embolization, surgical extirpation, or a combination of these modalities. Treatment and timing are often individualized to the patient and the extent of disease. For example, small-vessel AVMs are known to be localized and can be resected with good long-term outcomes [60]. Historically, young children were closely observed until disease expansion with the concept that the treatment should not be worse than the disease. However, this approach is currently being challenged due to the high recurrence rates experienced with AVM [61]. Diffuse lesions are a lifelong problem. Long-term followup with a dedicated multidisciplinary team is important for AVM management.

Intravascular embolization of AVM can be used alone or in combination with surgical excision. Absolute ethanol, polyvinyl alcohol, and ONYX have been employed as AVM embolization materials [62]. These agents selectively obstruct and destroy the arteries treated. Complications of this approach include local skin ulceration, soft tissue necrosis, mucosal sloughing, or nerve injury. Embolization provides temporary control of disease, but recurrence is high [61]. This is theoretically due to collateralization and recruitment of new vessels to support an undetected portion of the “nidus.” Frequent serial embolizations may improve patient outcomes.

In general, surgical management of AVMs requires preoperative supraselective embolization, judicious removal of tissue, and complex reconstructive techniques. In focal lesions, surgical excision has been shown to cure AVM [56, 63]. However, diffuse AVMs have recurrence rates as high as 93% [61]. Excision is preformed 24–48 hours after embolization. This helps control blood loss and define surgical margins of the lesion. Close postoperative observation with expected management of local recurrence is required. Recruitment of new vessels occurs after excision as well. In essence, AVMs are debilitating vascular malformations that are often misdiagnosed early in life. Despite successful initial therapy, these lesions may recur many years later making vigilant management necessary.

## 8. Conclusions

Vascular anomalies embody a myriad of blood vessels abnormalities that are thought to occur perinatally. Correct diagnosis is imperative for appropriate treatment. The most common vascular anomalies in order of presentation include hemangiomas, lymphatic malformations, capillary malformations (port-wine stains), venous malformations, and arteriovenous malformations. Treatment of vascular anomalies is complex and often involves multiple disciplines and therapeutic options. Referral to a vascular anomalies team is recommended when considering therapy for “problematic” hemangiomas and vascular malformations.

## Figures and Tables

**Table 1:** Classification of vascular anomalies.

Vascular tumors	Vascular malformations
	Slow-flow
Infantile hemangioma	Capillary malformations
Congenital hemangioma	Venous malformations
Tufted angioma	Lymphatic malformations
Kaposiform hemangioendothelioma	Fast-flow
	Arteriovenous malformations

**Table 2:** Old versus current nomenclature for describing hemangioma types.

Old nomenclature	New nomenclature
Strawberry or capillary hemangioma	Superficial hemangioma
Cavernous hemangioma	Deep hemangioma
Capillary cavernous hemangioma	Compound hemangioma
